# A Text-Book of Surgery

**Published:** 1902-02

**Authors:** 


A Text-Book of Surgery. By Dr. Hermann Tillmans, Professor in the University of Leipsic. Translated from the seventh German edition by Benjamin T. Tilton, M. D., and John Rogers, M. D., Instructors in Surgery, Cornell University. Edited by Lewis A. Stimson, M. D., Professor of Surgery, Cornell University. Volume I. The Principles of Surgery and Surgical Pathology. With 516 illustrations. Pp. viii.—841. New York: D. Appleton & Co. 1901.

This volume shows a general bringing to date of all the subjects treated in the first American edition. To chapter I. has been added a special paragraph on the sterilisation of the hands. Emphasis is laid upon Krönig's method. The author recommends gloves only as a means of protection in septic cases. In chapter II. a separate paragraph is given to Schleich's infiltration method for local anesthesia. No mention is made of intraspinal cocainisation. Under chapter VI. the sterilisation of catgut is fully considered. All of the standard methods are given. To chapter X. has been added a paragraph on the histological changes in skin grafting. In section 2, chapter I., paper wool is recommended as a dressing for wounds and under antiseptics, orthoform, tumenol and teucrin. A special paragraph on Créde's silver preparations and a paragraph on the treatment of eczema of the surgeon's hands. Section 3, chapter I., shows a number of additions. Under fungi are several new illustrations. The paragraph on the blastomycetes, which is present in the first American edition, 1894, at a time when these organisms were seldom mentioned in other works on surgery, is extended to include the recent observations of Busse and others, but does not include blastomycetic dermatitis, although the possible relation of these organisms to skin diseases is mentioned.

The paragraphs on bacteria are brought up to date, and that on toxins entirely rewritten. The paragraph on the mycetozoa and the mention of the plasmodiophora brassicæ, which is being so actively discussed in connection with cancer cannot fail to attract attention, inasmuch as this paragraph and the one following it on the protozoa appeared in the first American edition. The paragraphs on scar formation are based largely on the author's personal observation. The chapters on surgical diseases are excellent and that on diseases of bone is one of the best in any work on surgery. The chapter on tumors, especially the paragraphs dealing with the etiology of tumors, which takes cognisance of the work of Juergens on sarcoma, is excellent. The paragraph on the importance of microörganisms in the etiology of cancer reveals the author in an advanced position in this field.

Tillmans seems inclined toward the belief that the protozoa are the probable cause of cancer, although he considers that the cause has not been definitely established, inasmuch as the parasites in question have not yet been isolated nor carcinoma produced by inoculations of pure cultures. He observes, however, on the other hand, that the plasmodium of malaria has never been obtained in pure culture nor successfully inoculated upon man and that no one doubts that it is the parasite which causes malaria. Likewise, the coccidia are very sensitive bodies, very hard to inoculate upon animals and it seems that each specie can act as a parasite only in certain cells of a certain species of animals. Although the parasitic theory of carcinoma has not yet been proved, the author believes that since the recent investigations of Juergens the theory as very probable. He considers that in future investigation the warm stage microscope and fresh examination should be more largely employed.

On the whole the book shows that keen insight and close touch with the advance of pathology and surgery, which has given the previous editions of the work its just reputation. There is no work upon surgery in any language in which that portion dealing with surgical pathology is more in accord with the best teachings of the leading pathologists than the first volume of Tillmans's surgery. The work should emphasize the absolute necessity for the surgeon's being thoroughly acquainted with pathology, and it can be recommended as a most excellent guide for the student and a standard work of reference for the surgeon.—H. R. G.

